# Comments on: Association Study between Coronary Artery Disease and rs1333049 and rs10757274 Polymorphisms at 9p21 Locus in South-West Iran

**DOI:** 10.22074/cellj.2016.3849

**Published:** 2016-01-17

**Authors:** Preuß Michael H., Andreas Ziegler

**Affiliations:** 1 Institute of Medical Biometry and Statistics, University of Lübeck, University Medical Center Schleswig-Holstein, Campus Lübeck, Ratzeburger Allee 160, 23562 Lübeck, Germany; 2 Department of Pediatrics, University of Lübeck, University Medical Center Schleswig, Holstein, Campus Lübeck, Ratzeburger Allee 160, 23562 Lübeck, Germany; 3 Center for Clinical Trials, University of Lübeck, Ratzeburger Allee 160, 23562, Lübeck, Germany; 4 Nordic Center for Cardiovascular Research, Site Lübeck, Ratzeburger Allee 160, 23562 Lübeck, Germany; 5 School of Mathematics, Statistics and Computer Science, University of KwaZulu- Natal, Pietermaritzburg, South Africa

Foroughmand et al. (1) have recently reported association between coronary artery disease (CAD) and two well-known single nucleotide polymorphisms (SNPs) on chromosome 9p21.3 in subjects from South-West Iran. We doubt the validity of their findings.

Genotyping was done using ARMS-PCR for rs1333049 and rs10757274 in their study. When we first looked at the genotype frequencies, we observed a substantial excess of heterozygote subjects for both SNPs. Specifically, the relative excess of heterozygosity (REH) (2), a measure for the strength of deviation from Hardy-Weinberg equilibrium (HWE), was approximately 137% for rs1333049 in controls (REH=2.3688, Table 1). In contrast, we did not observe any deviation from HWE in our own studies (3, 4).

We additionally conducted a short literature search to identify other studies from Asia, which reported genotype frequencies in controls for rs1333049. These studies are summarized in table 1. None of these studies shows a deviation from HWE in their control groups (all P>0.05). In summary, only the recent study by Foroughmand and colleagues (1) shows a marked deviation from HWE in controls with this deviation observed for both reported SNPs.

Possible reasons for deviations from HWE have been summarized, e.g., in Ziegler et al. (2). The most likely cause for such a strong deviation from HWE is genotyping errors, especially because genotyping by ARMS-PCR plus gel electrophoresis is prone to such errors. However, REH could also be caused by population specifics, which has been discussed by Namipashaki et al. (5).

In any case, we (2) and others (5) recommend the investigation of HWE in population-based genetic association studies to improve quality and reliability of the research results.

**Table 1:** Genotype counts in control subjects together with relative excess of heterozygosity (REH), its confidence interval (95%-CI) and two-sided P values for rs1333049 as reported in several studies on Asian populations


Origin of population	Genotype counts in controls			REH	95%-CI	P value
	CC	CG	GG			

South-West Iran (1)	25	67	8	2.3688	[1.4886; 3.7694]	0.0003
Turkey (6)	85	115	40	0.9861	[0.7587; 1.2817]	0.9167
Japan I (7)	592	1204	636	0.9811	[0.9061; 1.0623]	0.6379
Japan II (8)	259	606	286	1.1133	[0.9916; 1.2499]	0.0692
Korea (8)	161	353	192	1.0039	[0.8659; 1.1638]	0.9591
Pakistan (9)	674	1290	609	1.0067	[0.9318; 1.0877]	0.8646

